# Puerarin attenuates diabetic kidney injury through interaction with Guanidine nucleotide‐binding protein Gi subunit alpha‐1 (Gnai1) subunit

**DOI:** 10.1111/jcmm.17414

**Published:** 2022-06-09

**Authors:** Qingqing Zhu, Shumin Yang, Chengguo Wei, Geming Lu, Kyung Lee, John Cijiang He, Ruijie Liu, Yifei Zhong

**Affiliations:** ^1^ Division of Nephrology Longhua Hospital, Shanghai University of Traditional Chinese Medicine Shanghai China; ^2^ Department of Medicine, Division of Nephrology Icahn School of Medicine at Mount Sinai New York New York USA; ^3^ Division of Endocrinology, Diabetes and Bone Diseases, Icahn School of Medicine at Mount Sinai Diabetes, Obesity and Metabolism Institute New York New York USA; ^4^ Icahn School of Medicine at Mount Sinai Mindich Child Health and Development Institute New York New York USA

**Keywords:** cAMP, CREB, diabetic kidney disease, Gnai1, podocyte, Puerarin

## Abstract

*Radix puerariae*, a traditional Chinese herbal medication, has been used to treat patients with diabetic kidney disease (DKD). Our previous studies demonstrated that puerarin, the active compound of *radix puerariae*, improves podocyte injury in type 1 DKD mice. However, the direct molecular target of puerarin and its underlying mechanisms in DKD remain unknown. In this study, we confirmed that puerarin also improved DKD in type 2 diabetic *db/db* mice. Through RNA‐sequencing odf isolated glomeruli, we found that differentially expressed genes (DEGs) that were altered in the glomeruli of these diabetic mice but reversed by puerarin treatment were involved mostly in oxidative stress, inflammatory and fibrosis. Further analysis of these reversed DEGs revealed protein kinase A (PKA) was among the top pathways. By utilizing the drug affinity responsive target stability method combined with mass spectrometry analysis, we identified guanine nucleotide‐binding protein Gi alpha‐1 (Gnai1) as the direct binding partner of puerarin. Gnai1 is an inhibitor of cAMP production which is known to have protection against podocyte injury. In vitro, we showed that puerarin not only interacted with Gnai1 but also increased cAMP production in human podocytes and mouse diabetic kidney in vivo. Puerarin also enhanced CREB phosphorylation, a downstream transcription factor of cAMP/PKA. Overexpression of CREB reduced high glucose‐induced podocyte apoptosis. Inhibition of PKA by Rp‐cAMP also diminished the effects of puerarin on high glucose‐induced podocyte apoptosis. We conclude that the renal protective effects of puerarin are likely through inhibiting Gnai1 to activate cAMP/PKA/CREB pathway in podocytes.

## 1 INTRODUCTION

Diabetic kidney disease (DKD) remains the leading cause of end‐stage kidney disease (ESKD) worldwide^1^, ^2^ and its incidence has also been increasing in China due to lifestyle changes,^3^, ^4^ With the recent introduction of sodium‐glucose cotransporter 2 inhibitors (SGLT2i), the combination therapy of RAS blockers and SGLT2i is considered the new standard therapy for DKD patients.^5^ However, many patients on these dual treatments continue to progress to ESKD,^6^ highlighting an as yet unmet need for more potent and safe therapies for DKD.

Podocyte injury, a major contributor to the development of microalbuminuria in DKD, occurs during the early stage of the disease,^7^ and the reduction in podocyte density predicts the progression of DKD.^8^, ^9^ In comparison with many pathways and molecular mechanisms that mediate podocyte injury in DKD (e.g. oxidative stress)^10^, ^11^ far fewer pathways have been defined to be protective against podocyte injury. Among such protective pathways is the activation of the cAMP/PKA pathway in podocytes by G protein‐coupled receptors (GPCR),^12^, ^13^ which help maintain podocyte morphology, actin assembly and matrix production.^12^, ^14^ In addition, cAMP is thought to attenuate the effect of hormones that activate the Ca^2+^/PKC pathway to maintain normal podocyte function.^15^ In support of the protective effects of cAMP/PKA in podocyte injury, we previously showed that all‐trans retinoic acid (atRA) attenuates HIV‐induced injury in part through enhanced cAMP production and PKA activity, leading to CREB phosphorylation^16^, ^17^ and that rolipram, an inhibitor of PDE4 that block the breakdown of cAMP, further enhanced the protective effects of atRA in podocytes.^18^ These studies firmly support the activation of the cAMP/PKA/CREB axis as a protective pathway in podocytes.

Although traditional Chinese herbal medications have been used extensively in Asia to treat patients with chronic kidney disease, the mechanisms of renoprotection have not been characterized in detail. Recently, we reported that arctigenin attenuates DKD through the PP2A phosphatase in podocytes.^19^ Thus, in the present study, we employed similar approaches to interrogate the cellular and molecular mechanisms of puerarin in DKD. We previously reported that Chen's Tangshen decoction, in which puerarin is a major component, is able to significantly reduce microalbuminuria in patients with early DN.^20^ This is further supported by a meta‐analysis that showed an additive effect in albuminuria reduction in DKD patients when puerarin was combined with ACE inhibitor.^21^ We and others have also shown that puerarin reduced albuminuria and kidney injury in several DKD models of type 1 diabetes.^22^, ^23^, ^24^ Several other studies have also demonstrated that puerarin has an anti‐oxidative effect.^22^, ^23^, ^25^, ^26^ We also demonstrated that puerarin attenuates DKD injury through regulation of Sirt1, heme oxygenase 1 and NOX4 in podocytes in DKD.^27^, ^28^ However, since these may be further downstream of puerarin's primary effects in podocytes, we employed an unbiased approach to further dissect the molecular mechanisms of puerarin by focusing on its direct binding partners. One of the direct interactors of puerarin identified was Gnai1, and our data suggest that puerarin attenuates podocyte injury through the inhibition of Gnai that results in the enhanced activation of cAMP/PKA pathway.

## 2 MATERIALS AND METHODS

### 2.1 Mouse studies

Male *db/db* mice in BKS background were purchased from The Jackson Laboratory (Bar Habor, ME). Sex‐ and age‐matched *db/m* littermates were used as controls. Puerarin (Sigma‐Aldrich, Saint Louis, MO) dissolved in 5% DMSO was administered in 10‐week‐old mice by oral gavage (20 mg/kg body weight/day) for 6 weeks. Vehicle‐treated mice were used as controls. All experimental methods were performed in accordance with the approved guidelines by the Institutional Animal Care and Use Committee at the Icahn School of Medicine at Mount Sinai, New York, NY.

### 2.2 Urine albumin and creatinine measurement

Urine albumin was measured using the ELISA kit (Bethyl Laboratory, Houston, TX), and urine creatinine was measured using a colorimetric assay kit (Cayman, Ann Arbor, MI).

### 2.3 Kidney histology

Kidneys were removed and fixed with 4% paraformaldehyde 16 h at 4°C. 4 μm sections were cut from paraffin‐embedded kidney tissues. Sections were stained with periodic acid–Schiff (PAS) for histology analysis. Assessment of the mesangial and glomerular cross‐sectional areas was performed by pixel counts on the kidney section in a blinded fashion, under 400x magnification (Zeiss AX10 microscope, Carl Zeiss Canada Ltd, Toronto, ON, Canada) as previously described.^29^, ^30^ Briefly, digitized images were scanned and profile areas were traced using ImageJ. The mean glomerular tuft volume was determined from the mean glomerular cross‐sectional area by light microscopy. The glomerular cross‐sectional area was calculated based on the average area of 30 glomeruli in each group, and glomerular tuft volume was calculated using the following equation:

$$\mathrm{GV}=\left(\beta /\kappa \right)\times {\mathrm{GA}}^{3/2}$$

where *β* = 1.38, the shape coefficient of spheres (the idealized shape of glomeruli), and *κ* = 1.1, the size distribution coefficient, and GA, glomerular area. Mesangial expansion was defined as a periodic acid–Schiff‐positive and nuclei‐free area in the mesangium. Quantification of mesangial expansion was based on 20 glomeruli cut at the vascular pole per mouse in each group.

### 2.4 RNA‐sequencing

Mice glomeruli were isolated as previously reported.^31^ Total RNA was extracted from glomeruli using Trizol method. Purified RNA underwent DNA digestion using RNase‐free DNase set (79,254, Qiagen). RNA‐sequencing was performed at Beijing Genomics Institute.

### 2.5 Cell culture

Immortalized human podocytes were obtained from Dr. Moin Saleem and cultured as described.^32^ Cells were serum‐starved in 1% serum‐containing medium for 12 h followed by treatment with the medium containing either normal glucose (5 mM, with 25 mM mannitol as high osmolarity control) or high glucose (30 mM) for the indicated time intervals. Rp‐cAMP was purchased from Sigma‐Aldrich (Cat: A165).

### 2.6 Drug affinity responsive target stability (DARTS)

DARTS was conducted as described previously.^29^, ^30^, ^33^ Briefly, podocytes were lysed in M‐PER mammalian protein extraction buffer (78,501 ThermoFisher) with proteinase inhibitor (11,836,153,001, Roche) and phosphatase inhibitors (50 mM NaF, 10 mM β‐glycerophosphate, 5 mM sodium pyrophosphate, 2 mM Na_3_VO_4_). 10X TNC buffer (500 mM Tris–HCl pH 8.0, 500 mM NaCl, 100 mM CaCl_2_) was added to the cell lysate and protein concentration was measured using the Bradford assay (Bio‐rad, #500–0006). The cell lysate was incubated with ATG at indicated concentrations at room temperature for 1 h. After incubation, cell lysates were subject to proteolysis with various concentrations of pronase (Roche, #10165921001) at room temperature for 20 min. Lysates were then submitted for mass spectrometry analysis or Western blot analysis.

### 2.7 Apoptosis assay

Differentiated podocytes cultured with normal glucose (Glucose 5.5 mM, Mannitol 24.5 mM) and high glucose (Glucose 30 mM) were treated with DMSO or puerarin for 24 h. Apoptosis was measured using the Annexin‐V FITC apoptosis detection kit (BD Bioscience) using flow cytometry.

### 2.8 Podocyte transfection

Podocytes were transfected with 5 μg of empty vector or corresponding vectors as indicated in the figures using Viafect reagent (E4981; Promega, Madison, WI) according to the manufacturer's protocol.

### 2.9 Western blot

Cells were lysed in M‐PER mammalian protein extraction reagent (ThermoFisher, Waltham, MA) containing protease and phophastase inhibitor cocktail. Protein was separated on SDS‐PAGE and transferred to PVDF membranes. Proteins were detected using specific antibodies: Gia1 (cell signalling 5290 1;1000 dilution), phosphor‐CREB (cell signalling 9190), total CREB (cell signalling 9197) and GAPDH (cell signalling 5174). The anti‐rabbit secondary antibody was obtained from Promega (W4018B) and used at 1:3000 dilution.

### 2.10 Measurement of Intracellular cAMP

This was measured using a cAMP Biotrak enzyme immunoassay system (Amersham Biosciences, Piscataway, NJ, USA). Cells were lysed, and 100 μl of cell lysates were used for the assay. A nonacetylation enzyme immunoassay procedure was used to measure intracellular cAMP production with a standard curve in a range from 12.5 to 3200 fmol/well. Similarly, glomerular lysates were used for the measurement of cAMP using the same method.

### 2.11 Statistical analysis

Data are expressed as mean ± SEM. The unpaired *t*‐test was used to comparison between groups or one‐way anova followed by Bonferroni correction was used when comparing between groups for treatment conditions using the GraphPad Prism software. *p‐*value <0.05 was considered statistically significant.

## 3 RESULTS

### 3.1 Puerarin attenuated proteinuria and glomerular injury in *db/db* mice

**FIGURE 1:**
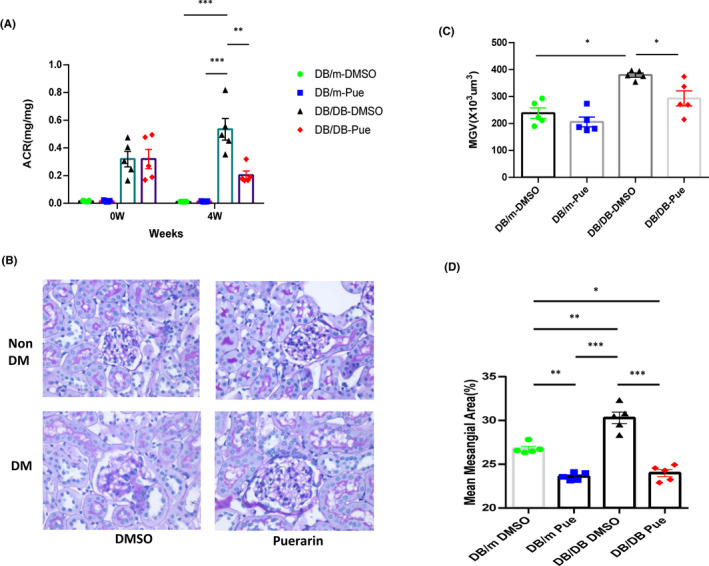
Puerarin treatment mitigates proteinuria and glomerular injury in diabetic db/db mice. The mice db/db mice at 10 weeks of age were given puerarin dissolved in 5% DMSO by oral gavage at a dose of 20 mg/kg body weight/day, or 5% DMSO vehicle as control, for 6 weeks. (A) Analysis of urinary albumin‐to‐creatinine ratio (UACR), *n* = 6 per group. (B) Representative images of periodic acid–Schiff (PAS)‐stained kidneys. Scale bar, 20 μm. (C and D) Quantification of the glomerular area and mesangial area fraction in diabetic and control eNOS^−/−^ mice, n = 6 per group. ****p < 0.0001 when compared to non‐diabetic controls, ^####^ *p* < 0.0001 when compared to vehicle‐treated diabetic eNOS^−/−^ mice by 2‐way anova with Tukey's post hoc analysis. The data are represented as mean ± SD. *p < 0.05; **p < 0.01 and ***p < 0.001

To examine whether puerarin can attenuate DKD, *db/db* and non‐diabetic control *db/m* mice were given either vehicle or puerarin (20 mg/kg) for 6 weeks, starting 10 weeks of age when albuminuria is evident in the *db/db* mice. Consistent with the previous results in the streptozotocin‐induced type 1 diabetic mice, puerarin treatment markedly attenuated diabetes‐induced albuminuria (Figure 1A). Glomerular injury as characterized by glomerular hypertrophy and mesangial expansion was also significantly attenuated by puerarin treatment (Figure 1B–D), indicating puerarin attenuates DKD in both type 1 and type 2 diabetic mice.

### 3.2 RNA‐sequencing shows puerarin‐induced gene expression changes in glomeruli of *db/db* mice

**FIGURE 2:**
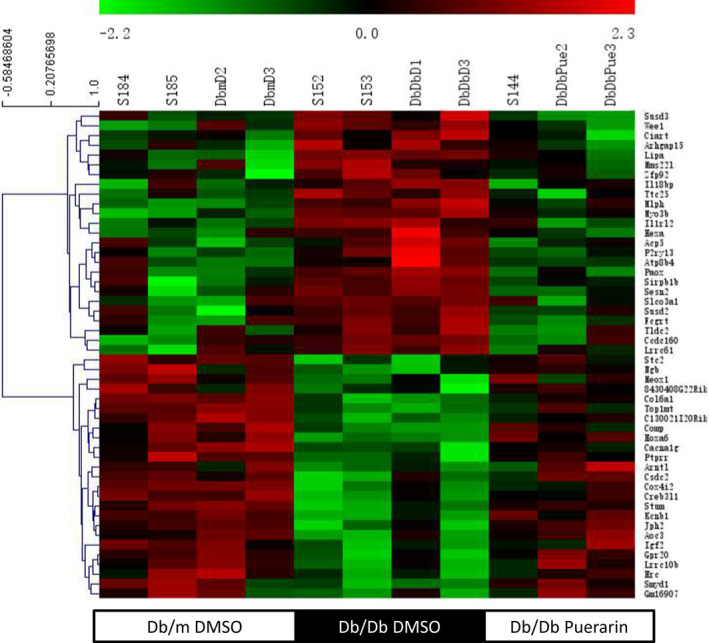
RNA‐seq data from isolated mouse glomeruli of diabetic db/db and control db/m mice with or without treatment of puerarin. RNA‐sequencing was performed with isolated glomeruli from diabetic (db/db, *n* = 4) or control (db/m, *n* = 4) mice treated with puerarin or vehicle (*n* = 3). Heat Map showing the number of differentially expressed genes (DEGs) in the diabetic mice that were upregulated in diabetic mice but downregulated by puerarin treatment

To elucidate the underlying mechanism of renoprotection conferred by puerarin in DKD, we performed the RNA‐sequencing of isolated glomeruli from the diabetic and control *db/db* mice treated with puerarin or vehicle. Figure 2 shows the heatmap of the top differentially expressed genes (DEGs) in the diabetic mice that were reversed by puerarin treatment. Gene enrichment analysis using the GO Biological Process showed that the regulation of response to wounding (fibrosis), oxidative stress and inflammation were the major pathways that were enriched by upregulated DEGs (db/db vs db/m) that were reversed by puerarin treatment (Table 1). The pathway analysis revealed that the fibrosis pathway was highly enriched in db/db mice vs db/dm mice and reversed by puerarin treatment (Table 2). In addition, several pathways known for DKD such as HIF1a, AMPK, Wnt/b‐catenin, TGF‐b, p38 MAPK and Rho pathways were also ranked highly in the list. Notably, protein kinase A (PKA) was among the top pathways which were reversed by puerarin treatment (Table 2).

**TABLE 1:** Top GO biological processes of differentially expressed genes(DEGs) in glomeruli of diabetic mice versus controls and of DEGs that are reversed by Puerarin treatment

Upregulated GO terms db/db vs db/m	
GO Term	*p*‐value
GO:0031667 ~ response to nutrient levels	5.09E−06
GO:0009611 ~ response to wounding	5.26E−06
GO:0006954 ~ inflammatory response	7.07E−06
GO:0007242 ~ intracellular signalling cascade	8.09E−06
GO:0048545 ~ response to steroid hormone stimulus	1.10E−05
GO:0009725 ~ response to hormone stimulus	1.12E−05
GO:0051384 ~ response to glucocorticoid stimulus	1.40E−05
GO:0007584 ~ response to nutrient	1.45E−05
GO:0009719 ~ response to endogenous stimulus	2.51E−05
GO:0031960 ~ response to corticosteroid stimulus	2.83E−05
GO:0070482 ~ response to oxygen levels	3.47E−05
GO:0001501 ~ skeletal system development	4.07E−05
GO:0048878 ~ chemical homeostasis	5.66E−05
GO:0006643 ~ membrane lipid metabolic process	6.54E−05
GO:0001666 ~ response to hypoxia	6.64E−05
GO:0048771 ~ tissue remodelling	7.11E−05
GO:0051050 ~ positive regulation of transport	9.68E−05

Downregulated GO terms db/db+puerarin vs db/db+vehicle	
GO Term	*p*‐value
GO:0055114 ~ oxidation reduction	0.000819
GO:0045580 ~ regulation of T cell differentiation	0.004328
GO:0009611 ~ response to wounding	0.006518
GO:0045619 ~ regulation of lymphocyte differentiation	0.008908
GO:0019221 ~ cytokine‐mediated signalling pathway	0.013237
GO:0050870 ~ positive regulation of T cell activation	0.015061
GO:0016042 ~ lipid catabolic process	0.0255
GO:0007596 ~ blood coagulation	0.026219
GO:0050817 ~ coagulation	0.026219
GO:0051251 ~ positive regulation of lymphocyte activation	0.028187
GO:0007599 ~ haemostasis	0.030233
GO:0007243 ~ protein kinase cascade	0.03236
GO:0042060 ~ wound healing	0.034312
GO:0002696 ~ positive regulation of leukocyte activation	0.035302
GO:0050867 ~ positive regulation of cell activation	0.039175
GO:0050863 ~ regulation of T cell activation	0.041599
GO:0048511 ~ rhythmic process	0.049313

**TABLE 2:** Top Pathways analysis using differentially expressed genes (DEGs) in glomeruli of diabetic mice versus controls and of DEGs that are reversed by Puerarin treatment

Top Pathways db/db vs db/m	
Ingenuity Canonical Pathways	‐log (*p*‐value)
Hepatic Fibrosis/Hepatic Stellate Cell Activation	5.76
GADD45 Signalling	4.97
Hereditary Breast Cancer Signalling	4.86
Cyclins and Cell Cycle Regulation	4.56
Axonal Guidance Signalling	4.56
Colorectal Cancer Metastasis Signalling	4.3
HIF1α Signalling	4.26
Amyotrophic Lateral Sclerosis Signalling	4.14
Glioblastoma Multiforme Signalling	4.1
Adipogenesis pathway	3.94
Bladder Cancer Signalling	3.9
Cell Cycle: G1/S Checkpoint Regulation	3.77
AMPK Signalling	3.63
Wnt/β‐catenin Signalling	3.61
Human Embryonic Stem Cell Pluripotency	3.53
Ovarian Cancer Signalling	3.49
TGF‐β Signalling	3.43
Tight Junction Signalling	3.4
Role of Macrophages, Fibroblasts and Endothelial Cells in Rheumatoid Arthritis	3.4

Top Pathways db/db+puerarin vs db/db+vehicle	
Ingenuity Canonical Pathways	‐log (*p*‐value)
Hepatic Fibrosis/Hepatic Stellate Cell Activation	5.43
p38 MAPK Signalling	3.47
Regulation of Actin‐based Motility by Rho	3.04
ILK Signalling	2.8
Phenylethylamine Degradation I	2.54
Cardiac Hypertrophy Signalling	2.15
3‐phosphoinositide Degradation	2.05
Cellular Effects of Sildenafil (Viagra)	2.03
Integrin Signalling	1.98
Growth Hormone Signalling	1.97
Agranulocyte Adhesion and Diapedesis	1.96
Nicotine Degradation II	1.88
Germ Cell‐Sertoli Cell Junction Signalling	1.8
Superpathway of Inositol Phosphate Compounds	1.78
Protein Kinase A Signalling	1.77
Bupropion Degradation	1.75
Ethanol Degradation IV	1.75
Calcium Signalling	1.71
Pregnenolone Biosynthesis	1.7

### 3.3 Gnai1 is a direct target protein of puerarin in kidney cells

**FIGURE 3:**
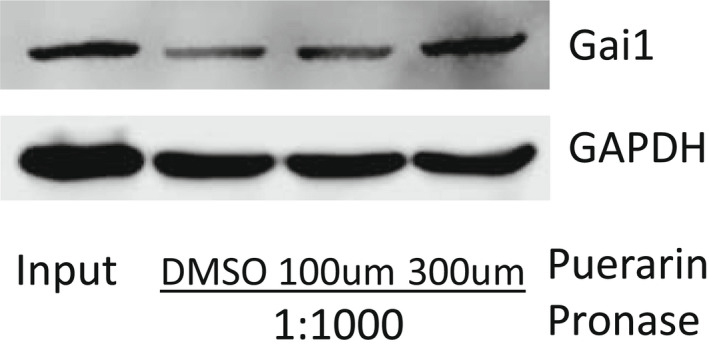
ATG binds to Gnai1. Drug Affinity Responsive Target Stability (DARTS) assay was performed to test the direct binding of puerarin to Gnai1 in podocytes. (A) Cell lysates were pre‐incubated with various concentrations of puerarin as indicated at 25°C for 1 h prior to digestion with pronase (0 or 1:1000 dilution) for 20 min. Lysates were then probed for Gnai1 expression, with GAPDH as a loading control

**FIGURE 4:**
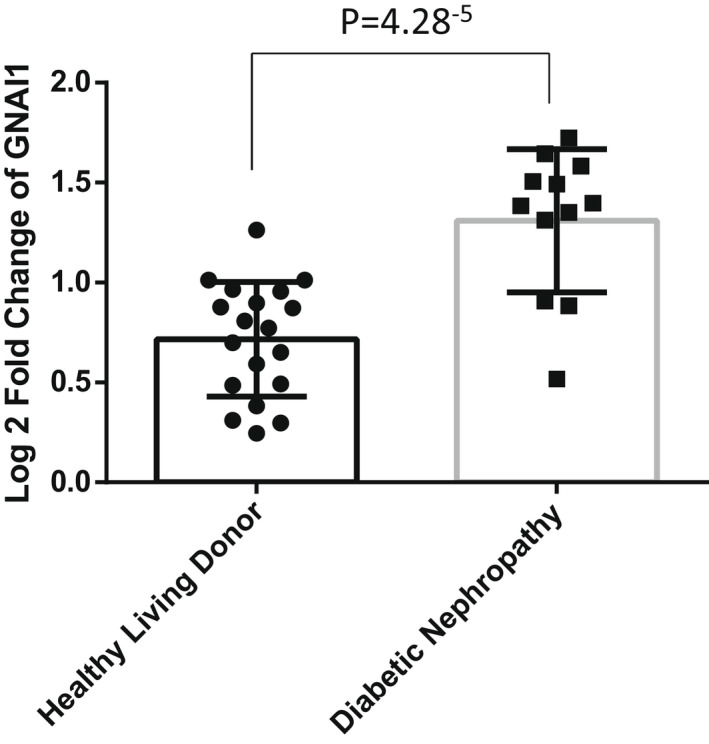
Expression of Gnai1 mRNAs in human kidneys. The dataset obtained from Nephroseq (nephroseq.org) show the expression levels of Gia1 increases in the glomeruli of kidneys from patients with diabetic kidney disease (DKD) compared with healthy living donors (HLD)

To determine the direct binding partner of puerarin in podocytes, we next utilized the drug affinity responsive target stability (DARTS) method.^29^, ^30^, ^33^ DARTS assay is based on the principle that the binding of a small molecule compound to the target protein changes the protein conformation, leading to increased or decreased protein stability and protection against proteolysis. To analyse the puerarin‐bound proteins, mass spectrometry analysis was performed following the DARTS assay using pronase digestion in podocytes treated with vehicle or puerarin. Table 3 shows the list of the top 15 puerarin‐bound proteins. While the top and third puerarin‐bound proteins were keratin, a highly abundant cytoskeletal protein that may have non‐specific interactions with puerarin, the second protein on the list was the Guanine nucleotide‐binding protein G_i_ subunit alpha 1 (Gnai1), a member of the G protein family. G_i_ proteins primarily mediate the inhibition of the cAMP‐dependent pathway by inhibiting adenylyl cyclase activity.^34^ However, the role of Gnai1 has never been studied in the context of kidney disease. The other proteins in the list might be also important in mediating the effects of puerarin in podocytes such as Exosome complex component RRP41 and NADH–ubiquinone oxidoreductase 75 kDa subunit but these will be investigated in the future studies. The interaction of puerarin with Gnai1 was confirmed by Western blot following the DARTS assay (Figure 3A). Interestingly, glomerular expression of *GNAI1* transcript was increased in human DKD as compared to healthy living donors^35^ (Figure 4).

**TABLE 3:** Puerarin‐interacting proteins identified from DARTS followed by mass spectrometry

Identified proteins	Accession number	Molecular weight	Control spectra counts	Puerarin spectra counts	Puerarin/control ratio
Keratin, type I cytoskeletal 13 OS = Homo sapiens GN = KRT13 PE = 1 SV = 4	P13646	50 kDa	0	9	5.7
Guanine nucleotide‐binding protein G(i) subunit alpha‐1 OS = Homo sapiens GN = GNAI1 PE = 1 SV = 2	P63096	40 kDa	0	7	4.7
Keratin, type I cytoskeletal 15 OS = Homo sapiens GN = KRT15 PE = 1 SV = 3	P19012	49 kDa	0	7	4.3
Ras‐related C3 botulinum toxin substrate 3 OS = Homo sapiens GN = RAC3 PE = 1 SV = 1	P60763	21 kDa	0	6	3.8
Glutamine‐‐fructose‐6‐phosphate aminotransferase [isomerizing] 2 OS = Homo sapiens GN = GFPT2 PE = 1 SV = 3	O94808	77 kDa	0	5	3.3
Acyl‐protein thioesterase 2 OS = Homo sapiens GN = LYPLA2 PE = 1 SV = 1	O95372	25 kDa	0	4	2.9
Bifunctional 3′‐phosphoadenosine 5′‐phosphosulfate synthase 1 OS = Homo sapiens GN = PAPSS1 PE = 1 SV = 2	O43252	71 kDa	0	4	2.9
Dedicator of cytokinesis protein 7 OS = Homo sapiens GN = DOCK7 PE = 1 SV = 4	Q96N67	243 kDa	0	4	2.9
Endoribonuclease LACTB2 OS = Homo sapiens GN = LACTB2 PE = 1 SV = 2	Q53H82	33 kDa	0	4	2.9
Exosome complex component RRP41 OS = Homo sapiens GN = EXOSC4 PE = 1 SV = 3	Q9NPD3	26 kDa	0	4	2.9
Keratin, type II cytoskeletal 4 OS = Homo sapiens GN = KRT4 PE = 1 SV = 4	P19013	57 kDa	0	4	2.9
NADH–ubiquinone oxidoreductase 75 kDa subunit, mitochondrial OS = Homo sapiens GN = NDUFS1 PE = 1 SV = 3	P28331	79 kDa	0	4	2.9
NIF3‐like protein 1 OS = Homo sapiens GN = NIF3L1 PE = 1 SV = 2	Q9GZT8	42 kDa	0	4	2.9
Plasma membrane calcium‐transporting ATPase 4 OS = Homo sapiens GN = ATP2B4 PE = 1 SV = 2	P23634	138 kDa	0	4	2.9
Ras‐related protein Rab‐6B OS = Homo sapiens GN = RAB6B PE = 1 SV = 1	Q9NRW1	23 kDa	0	4	2.9
Regulatory‐associated protein of mTOR OS = Homo sapiens GN = RPTOR PE = 1 SV = 1	Q8N122	149 kDa	0	4	2.9
Unconventional myosin‐Ib OS = Homo sapiens GN = MYO1B PE = 1 SV = 3	O43795	132 kDa	0	4	2.9

### 3.4 cAMP/PKA/CREB mediates the effects of Puerarin in podocytes

**FIGURE 5:**
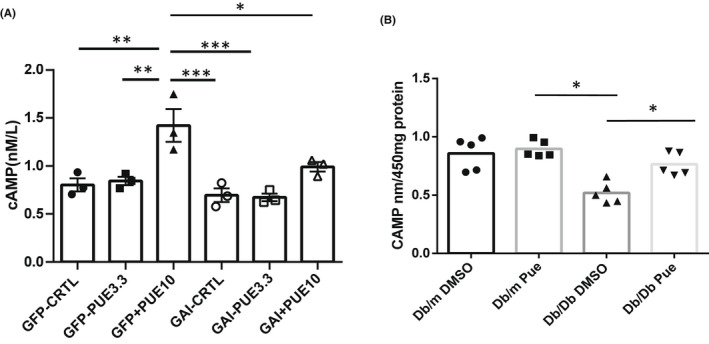
Puerarin stimulates cAMP production in podocytes. (A) Puerarin stimulated cAMP production through Gnai1. 10 μm of Puerarin treatment produced more cAMP in podocytes, which was abolished when there was GNAI1 overexpression. (B) Puerarin restored decreased cAMP levels in diabetic kidney cortex. *p < 0.05, **p < 0.01 and ***p < 0.005 between the groups, *n* = 3

**FIGURE 6:**
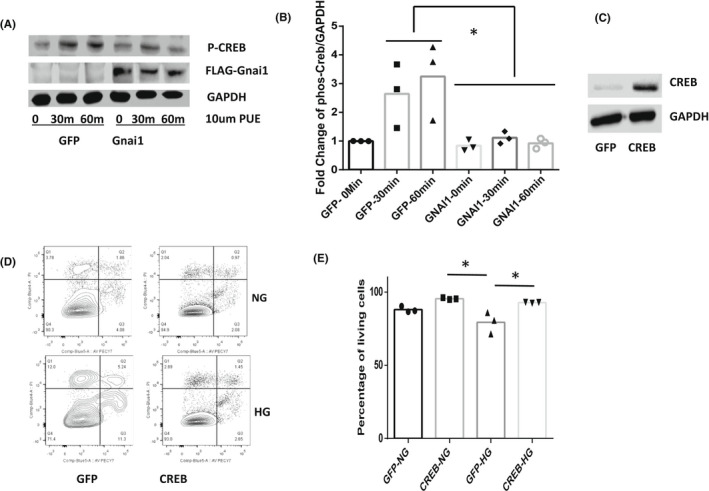
Puerarin increases phosphorylation of CREB through inhibiting Gia1 and CREB protects podocytes against apoptosis induced by high glucose. (A) Differentiated human podocytes were transfected with GFP or CREB tagged with FLAG and then were treated with or without 10 μm PUE for 30 and 60 min. Phos‐creb was increased with PUE treatment, while it was inhibited in CREB expressing cells. (B) Densitometric analysis of the Western blots shown in the Figure 6A. (C) CREB expression in podocytes. (D) Apoptosis was examined by flowcytometry in podocytes expressing GFP and CREB treated with or without 10 μm PUE for 24 h. (E) Quantification of living cells for each group. *p < 0.05, *n* = 3

**FIGURE 7:**
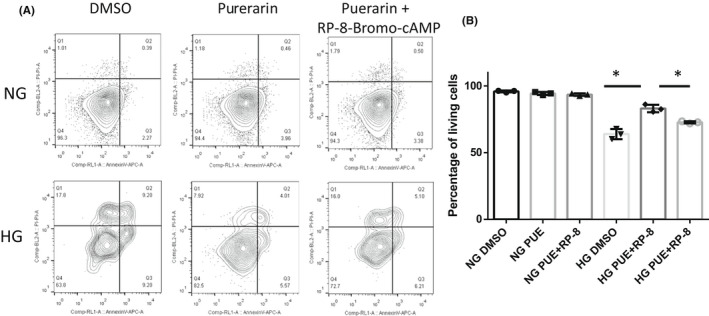
Inhibition of cAMP/PKA pathway by Rp‐cAMP diminishes protective effects of puerarin on podocyte apoptosis in high glucose condition. (A) Apoptosis was examined by flowcytometry in podocytes treated with normal glucose (NG) or high glucose (HG) together with or without 10 μm puerarin (PUE) or Rp‐cAMP (10 μm) for 24 h. (B) Quantification of living cells for each group. *p < 0.05, *n* = 3

In cultured podocytes, treatment of 10 μM puerarin increased intracellular cAMP production, which was inhibited by overexpression of Gnai1 (Figure 5A). Moreover, cAMP measurement showed reduced cAMP levels in isolated glomeruli from *db/db* mice compared with *db/m* mice, but restored levels in the glomeruli of db/db mice treated with puerarin (Figure 5B). In addition, puerarin induced CREB phosphorylation, which was reduced by Gnai1 overexpression (Figure 6A). Moreover, CREB overexpression inhibited high glucose‐induced podocyte apoptosis (Figure 6B–C). In addition, we showed that inhibition of PKA activation by using a cAMP antagonist, Rp‐cAMP, diminished the protective effects of puerarin on high glucose‐induced podocyte apoptosis (Figure 7). Together, our data indicate that puerarin protects podocytes in diabetic kidneys by interacting with Gnai1, thereby enhancing the activation of cAMP/PKA/CREB pathway.

## 4 DISCUSSION

In the current study, we confirmed that puerarin mitigates albuminuria and kidney injury in db/db mice, a model of type 2 diabetes. Together with our previous study in type 1 diabetic mice^22^, ^27^, ^28^ our results provide strong evidence to support a therapeutic effect of puerarin for DKD. Puerarin has shown renoprotective effects in several animal studies^22^, ^23^, ^24^ and several pilot clinical studies suggest that puerarin treatment significantly reduced albuminuria in patients with stage 3 DN.^21^ However, larger randomized clinical trials are required to confirm its renoprotective effects in DKD patients. Puerarin, a major isoflavonoid component from the root of *pueraria candollei* of *Leguminosae* family, has a structure of 7‐hydroxy‐3‐(4‐hydroxyphenyl)‐1‐benzopyran‐4‐one‐8‐β‐D‐glucopyranoside.^36^ We believe that puerarin and its analogues could be developed as potential drugs to treat patients with DKD. Future studies are also required to determine whether puerarin could provide additional renoprotection in conjunction with ACEi/ARBs and SGLT2i.

**FIGURE 8:**
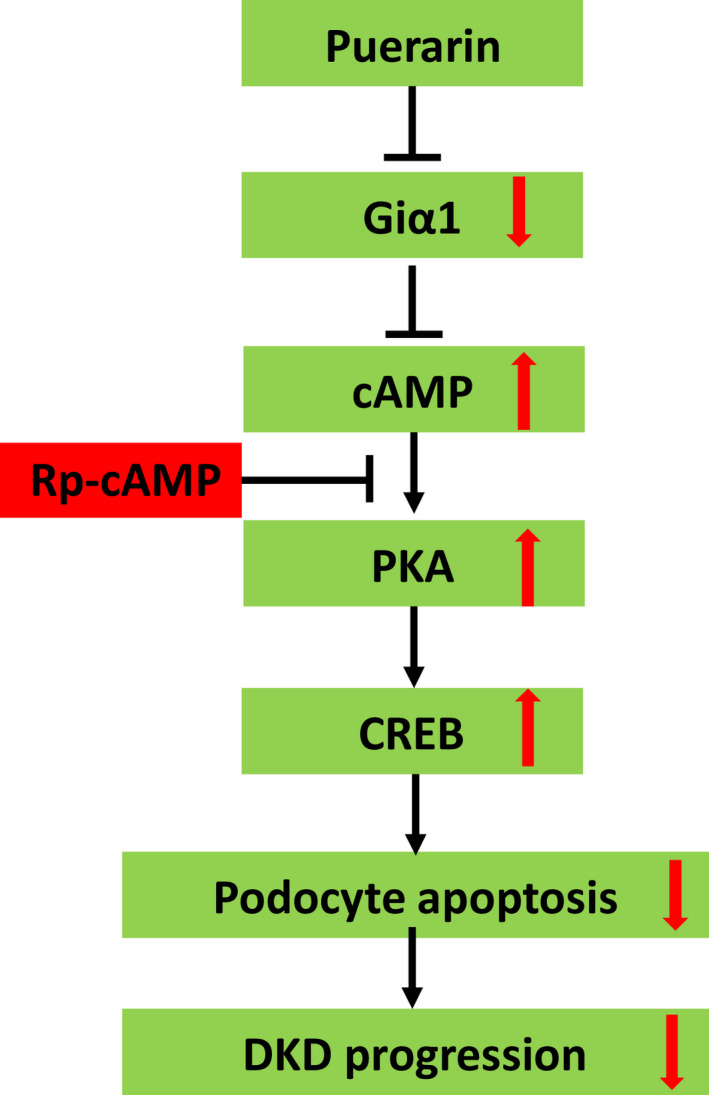
Schema of the summary for the renal protective effects of puerarin in DKD. Puerarin interacts with and inhibits Gia1 to stimulate cAMP/PKA pathway. Activation of cAMP/PKA pathway diminishes high glucose‐induced podocyte apoptosis

Several previous studies support an anti‐oxidative effect of puerarin in kidney disease^22^, ^23^, ^25^, ^26^ as well as in cardiovascular^37^ and neurological diseases.^38^ We previously reported that the anti‐oxidative effect of puerarin is in part mediated through the suppression of NOX4 expression in podocytes.^27^ In addition, our previous studies demonstrated that puerarin upregulates SIRT1 expression and induces autophagy in podocytes.^27^, ^28^ However, how puerarin regulates these downstream pathways was unclear. In addition, the direct interacting proteins of puerarin have never been identified in the kidney cells or others. To further dissect the mechanisms of action of puerarin in DKD, we took two approaches here as discussed below.

First, we performed unbiased screening of DEGs and pathways which are altered in DKD but reversed by puerarin treatment. This unbiased screening approach allowed us to verify not only the previous findings but also to identify additional novel mechanisms of puerarin in DKD. Importantly, we found that puerarin was able to reverse oxidative stress, inflammatory and fibrosis pathways in the DKD, further confirming a critical role of puerarin in attenuating major pathological processes in the DKD. We also found that puerarin regulates lipid metabolism pathway (Table 1) and the effect of puerarin in lipid metabolism has been also reported.^39^, ^40^ Interestingly, the top pathways reversed by puerarin was related to fibrosis (Table 2) and future studies are required to further determine the anti‐fibrosis effect of puerarin in kidney disease.

Second, we determined the direct binding partner of puerarin by using DARTS method^29^, ^30^, ^33^ and identified Gnai1 as a direct binding partner of puerarin in podocytes. Using a similar approach, we previously identified PP2A as a direct binding partner of arctigenin in podocytes to confer protection against DKD.^19^ Gnai1 exerts inhibitory effects on cAMP production through its interaction with GPCR and inactivation of adenyl cyclase.^41^ Although Gq signalling was shown to mediate the effects of TRPC6 in glomerular disease,^21^ the role of Gi has never been studied in the podocytes and in the context of kidney disease. However, several studies demonstrate a protective role of cAMP/PKA/CREB pathway in podocytes.^12^, ^14^, ^16^ Therefore, we hypothesized that puerarin interacts with Gia1 to inhibit its activity, which in turn results in the activation of cAMP/PKA/CREB pathway and podocyte protection. This was indeed demonstrated by the following evidence: (1) We confirmed the interaction between puerarin and Gnai1 by DARTS assay followed by Western blot; (2) Gnai1 expression is increased in the glomeruli of human DKD; (3) Puerarin increases cAMP production which was suppressed by overexpression of Gnai1 in podocytes; (4) Puerarin also induces CREB phosphorylation and overexpression of CREB reduces podocyte apoptosis which mimic the effects of puerarin; and (5) Inhibition of cAMP/PKA pathway attenuates the protective effects of puerarin on high glucose‐induced podocyte apoptosis. These studies also suggest a critical role of Gnai1 in the pathogenesis of DKD. Future studies are required to further confirm the role of Gnai1 in podocyte injury in DKD by using knockout mice.

How puerarin‐mediated activation of cAMP/PKA pathway links to its anti‐oxidative stress pathway in kidney cells remains to be determined. These two pathways could be activated in parallel. However, recent studies suggest that activation of cAMP/PKA pathway could have anti‐oxidative stress effects in renal tubular cells during ischemic reperfusion‐induced injury via activation of EPAC pathway.^42^ The studies also suggest that cAMP pathway mediates the renoprotective effects of GLP1R agonists in DKD and one of the mechanisms is anti‐oxidative stress.^43^

In summary, we confirmed the renoprotective effects of puerarin in a type 2 diabetic model with DKD. By RNA‐sequencing of glomeruli from these mice, we confirmed that puerarin has anti‐oxidative stress, anti‐inflammatory and anti‐fibrosis effects. We identified Gnai1 as a direct binding partner of puerarin in podocytes and demonstrated that puerarin activates cAMP/PKA/CREB pathway to protect podocytes from injury via inhibition of Gnai1 (Figure 8). Our study reveals a novel renal protective mechanism of puerarin and further support its therapeutic role for patients with DKD.

## AUTHOR CONTRIBUTIONS

**Qingqing Zhu:** Investigation (lead). **Shumin Yang:** Investigation (supporting). **Chengguo Wei:** Data curation (lead); formal analysis (lead); investigation (supporting). **Geming Lu:** Data curation (supporting); methodology (supporting). **Kyung Lee:** Data curation (supporting); investigation (supporting); writing – original draft (lead). **John Cijiang He:** Conceptualization (supporting); methodology (lead); project administration (supporting); supervision (supporting); writing – review and editing (supporting). **Ruijie Lu:** Formal analysis (supporting); project administration (supporting); validation (equal); writing – original draft (lead). **Yifei Zhong:** Conceptualization (lead); funding acquisition (lead); project administration (lead); validation (lead); writing – original draft (supporting); writing – review and editing (lead).

## CONFLICT OF INTEREST

The authors confirm that there are no conflicts of interest.

## Data Availability

The data that support the findings of this study are available from the corresponding author upon reasonable request.

## References

[1] USRDS . Annual Data Report: Atlas of End‐Stage‐Renal‐Disease in the United States. USRDS; 2011.

[2] World Health Organization . Global Status Report on Noncommunicable Diseases. World Health Organization; 2014.

[3] Zhang L , Long J , Jiang W , et al. Trends in chronic kidney disease in China. N Engl J Med. 2016;375:905‐906. 27579659 10.1056/NEJMc1602469

[4] Yang W , Lu J , Weng J , et al. Prevalence of diabetes among men and women in China. N Engl J Med. 2010;362:1090‐1101. 20335585 10.1056/NEJMoa0908292

[5] Alicic RZ , Johnson EJ , Tuttle KR . SGLT2 inhibition for the prevention and treatment of diabetic kidney disease: a review. Am J Kidney Dis. 2018;72:267‐277. 29866460 10.1053/j.ajkd.2018.03.022

[6] de Zeeuw D . Unmet need in renal protection‐‐do we need a more comprehensive approach? Contrib Nephrol. 2011;171:157‐160. 21625105 10.1159/000327337

[7] Ijpelaar DH , Schulz A , Koop K , et al. Glomerular hypertrophy precedes albuminuria and segmental loss of podoplanin in podocytes in Munich‐Wistar‐Fromter rats. Am J Physiol Renal Physiol. 2008;294:F758‐F767. 18199599 10.1152/ajprenal.00457.2007

[8] Meyer TW , Bennett PH , Nelson RG . Podocyte number predicts long‐term urinary albumin excretion in Pima Indians with type II diabetes and microalbuminuria. Diabetologia. 1999;42:1341‐1344. 10550418 10.1007/s001250051447

[9] Steffes MW , Schmidt D , McCrery R , Basgen JM . Glomerular cell number in normal subjects and in type 1 diabetic patients. Kidney Int. 2001;59:2104‐2113. 11380812 10.1046/j.1523-1755.2001.00725.x

[10] Susztak K , Böttinger E , Novetsky A , et al. Molecular profiling of diabetic mouse kidney reveals novel genes linked to glomerular disease. Diabetes. 2004;53:784‐794. 14988265 10.2337/diabetes.53.3.784

[11] Jha JC , Gray SP , Barit D , et al. Genetic targeting or pharmacologic inhibition of NADPH oxidase nox4 provides renoprotection in long‐term diabetic nephropathy. J Am Soc Nephrol. 2014;25:1237‐1254. 24511132 10.1681/ASN.2013070810 PMC4033375

[12] Endlich N , Endlich K . cAMP pathway in podocytes. Microsc Res Tech. 2002;57:228‐231. 12012389 10.1002/jemt.10079

[13] Bek M , Nusing R , Kowark P , et al. Characterization of prostanoid receptors in podocytes. J Am Soc Nephrol. 1999;10:2084‐2093. 10505684 10.1681/ASN.V10102084

[14] Faul C , Donnelly M , Merscher‐Gomez S , et al. The Actin cytoskeleton of kidney podocytes is a direct target of the antiproteinuric effect of cyclosporine a. Nat Med. 2008;14:931‐938. 18724379 10.1038/nm.1857 PMC4109287

[15] Endlich N , Nobiling R , Kriz W , Endlich K . Expression and signaling of parathyroid hormone‐related protein in cultured podocytes. Exp Nephrol. 2001;9:436‐443. 11702004 10.1159/000052643

[16] He JC , Lu TC , Fleet M , et al. Retinoic acid inhibits HIV‐1‐induced podocyte proliferation through the cAMP pathway. J Am Soc Nephrol. 2007;18:93‐102. 17182884 10.1681/ASN.2006070727 PMC3197239

[17] Nakagawa T , Sato W , Glushakova O , et al. Diabetic endothelial nitric oxide synthase knockout mice develop advanced diabetic nephropathy. J Am Soc Nephrol. 2007;18:539‐550. 17202420 10.1681/ASN.2006050459

[18] Zhong Y , Wu Y , Liu R , et al. Roflumilast enhances the renal protective effects of retinoids in an HIV‐1 transgenic mouse model of rapidly progressive renal failure. Kidney Int. 2012;81:856‐864. 22258322 10.1038/ki.2011.467 PMC3326224

[19] Zhong Y , Lee K , Deng Y , et al. Arctigenin attenuates diabetic kidney disease through the activation of PP2A in podocytes. Nat Commun. 2019;10:4523. 31586053 10.1038/s41467-019-12433-w PMC6778111

[20] lian S , Wang L , Zhang X , et al. Clinical investigation of stage‐IV diabetic nephropathy with treatment of Chen's Tangshen decoction and conventional Western medicine. Shanghai J Trad Chinese Med. 2010;44:3.

[21] Wang B , Chen S , Yan X , et al. The therapeutic effect and possible harm of puerarin for treatment of stage III diabetic nephropathy: a meta‐analysis. Altern Ther Health Med. 2015;21:36‐44. 25599431

[22] Zhong Y , Zhang X , Cai X , Wang K , Chen Y , Deng Y . Puerarin attenuated early diabetic kidney injury through down‐regulation of matrix metalloproteinase 9 in streptozotocin‐induced diabetic rats. PLoS One. 2014;9:e85690. 24454919 10.1371/journal.pone.0085690 PMC3893265

[23] Xu X , Zheng N , Chen Z , Huang W , Liang T , Kuang H . Puerarin, isolated from Pueraria lobata (Willd.), protects against diabetic nephropathy by attenuating oxidative stress. Gene. 2016;591:411‐416. 27317894 10.1016/j.gene.2016.06.032

[24] Zhang Y , Wang H , Yu L , Chen J . The Puerarin improves renal function in STZ‐induced diabetic rats by attenuating eNOS expression. Ren Fail. 2015;37:699‐703. 25707518 10.3109/0886022X.2015.1011500

[25] Ma JQ , Ding J , Xiao ZH , Liu CM . Puerarin ameliorates carbon tetrachloride‐induced oxidative DNA damage and inflammation in mouse kidney through ERK/Nrf2/ARE pathway. Food Chem Toxicol. 2014;71:264‐271. 24975872 10.1016/j.fct.2014.06.017

[26] Kim KM , Jung DH , Jang DS , et al. Puerarin suppresses AGEs‐induced inflammation in mouse mesangial cells: a possible pathway through the induction of heme oxygenase‐1 expression. Toxicol Appl Pharmacol. 2010;244:106‐113. 20060010 10.1016/j.taap.2009.12.023

[27] Li X , Cai W , Lee K , et al. Puerarin attenuates diabetic kidney injury through the suppression of NOX4 expression in podocytes. Sci Rep. 2017;7:14603. 29097815 10.1038/s41598-017-14906-8 PMC5668268

[28] Li X , Zhu Q , Zheng R , et al. Puerarin attenuates diabetic nephropathy by promoting autophagy in podocytes. Front Physiol. 2020;11:73. 32116781 10.3389/fphys.2020.00073 PMC7033627

[29] Pai MY , Lomanick B , Hwang H , et al. Drug affinity responsive target stability (DARTS) for small‐molecule target identification. Methods Mol Biol. 2015;1263:287‐298. 25618353 10.1007/978-1-4939-2269-7_22 PMC4442491

[30] Liu R , Das B , Xiao W , et al. A novel inhibitor of homeodomain interacting protein kinase 2 mitigates kidney fibrosis through inhibition of the TGF‐beta1/Smad3 pathway. J Am Soc Nephrol. 2017;28:2133‐2143. 28220029 10.1681/ASN.2016080841 PMC5491283

[31] Fu J , Wei C , Zhang W , et al. Gene expression profiles of glomerular endothelial cells support their role in the glomerulopathy of diabetic mice. Kidney Int. 2018;94:326‐345. 29861058 10.1016/j.kint.2018.02.028 PMC6054896

[32] Saleem MA , O’Hare MJ , Reiser J , et al. A conditionally immortalized human podocyte cell line demonstrating nephrin and podocin expression. J Am Soc Nephrol. 2002;13:630‐638. 11856766 10.1681/ASN.V133630

[33] Lomenick B , Hao R , Jonai N , et al. Target identification using drug affinity responsive target stability (DARTS). Proc Natl Acad Sci U S A. 2009;106:21984‐21989. 19995983 10.1073/pnas.0910040106 PMC2789755

[34] Kano H , Toyama Y , Imai S , et al. Structural mechanism underlying G protein family‐specific regulation of G protein‐gated inwardly rectifying potassium channel. Nat Commun. 2019;10:2008. 31043612 10.1038/s41467-019-10038-x PMC6494913

[35] Ju W , Nair V , Smith S , et al. Tissue transcriptome‐driven identification of epidermal growth factor as a chronic kidney disease biomarker. Sci Transl Med. 2015;7:316ra193. 10.1126/scitranslmed.aac7071 PMC4861144 26631632

[36] Duan HJ , Liu SX , Zhang YJ , Liu QJ , He N , Li YM . Effects of puerarin on renal function, expressions of MMP‐2 and TIMP‐2 in diabetic rats. Yao Xue Xue Bao. 2004;39:481‐485. 15493832

[37] Wu L , Qiao H , Li Y , Li L . Protective roles of puerarin and Danshensu on acute ischemic myocardial injury in rats. Phytomedicine. 2007;14:652‐658. 17870452 10.1016/j.phymed.2007.07.060

[38] Zhang H , Liu Y , Lao M , Ma Z , Yi X . Puerarin protects Alzheimer's disease neuronal cybrids from oxidant‐stress induced apoptosis by inhibiting pro‐death signaling pathways. Exp Gerontol. 2011;46:30‐37. 20933077 10.1016/j.exger.2010.09.013

[39] Zheng G , Lin L , Zhong S , Zhang Q , Li D . Effects of puerarin on lipid accumulation and metabolism in high‐fat diet‐fed mice. PloS One. 2015;10:e0122925. 25822741 10.1371/journal.pone.0122925 PMC4378957

[40] Chen T , Cao Q , Wang Y , Harris DCH . M2 macrophages in kidney disease: biology, therapies, and perspectives. Kidney Int. 2019;95:760‐773. 30827512 10.1016/j.kint.2018.10.041

[41] Stojilkovic SS , Murano T , Gonzalez‐Iglesias AE , et al. Multiple roles of Gi/o protein‐coupled receptors in control of action potential secretion coupling in pituitary lactotrophs. Ann N Y Acad Sci. 2009;1152:174‐186. 19161388 10.1111/j.1749-6632.2008.03994.x PMC2733166

[42] Stokman G , Qin Y , Booij TH , et al. Epac‐rap signaling reduces oxidative stress in the tubular epithelium. J Am Soc Nephrol. 2014;25:1474‐1485. 24511123 10.1681/ASN.2013070679 PMC4073429

[43] Galindo RJ , Pasquel FJ , Vellanki P , et al. Degludec hospital trial: a randomized controlled trial comparing insulin degludec U100 and glargine U100 for the inpatient management of patients with type 2 diabetes. Diabetes Obes Metab. 2022;24:42‐49. 34490700 10.1111/dom.14544 PMC8665002

